# (*S*)-2,2′-Dihy­droxy-*N*,*N*′-(6-hy­droxy­hexane-1,5-di­yl)dibenzamide

**DOI:** 10.1107/S1600536813000354

**Published:** 2013-01-12

**Authors:** Sabine Wilbrand, Christian Neis, Kaspar Hegetschweiler

**Affiliations:** a Fachrichtung Chemie, Universität des Saarlandes, Postfach 151150, D-66041 Saarbrücken, Germany

## Abstract

In the title compound, C_20_H_24_N_2_O_5_, the dihedral angle between the two roughly planar salicyl­amide fragments [r.m.s. deviations = 0.043 (2) and 0.149 (2) Å] is 25.50 (5)°. The mol­ecular conformation is stabilized by intra­molecular O—H⋯O hydrogen bonds involving phenol –OH groups and amide O atoms. Inter­molecular hy­droxy­meth­yl–amide O—H⋯O and amine–hy­droxy­methyl N—H⋯O hydrogen bonds form infinite chains along the *b* axis. These chains are further inter­linked by amine–amide N—H⋯O and phenol–phenol O—H⋯O inter­actions, thus giving layers parallel to (001).

## Related literature

For the isolation and physico-chemical properties of myxochelin A, see: Kunze *et al.* (1989 ▶). For the crystal structure of *N*,*N*′-(pentane-1,5-di­yl)bis­(3-meth­oxy­salicyl­amide), see: Huang *et al.* (1995 ▶). For metal complex formation with linear bis-catechol amides and linear bis-salicyl­amides, see: Duhme *et al.* (1996 ▶); Huang *et al.* (1995 ▶); Cappillino *et al.* (2009 ▶); Stoicescu *et al.* (2009 ▶). For the treatment of H atoms in *SHELXL*, see: Müller *et al.* (2006 ▶).
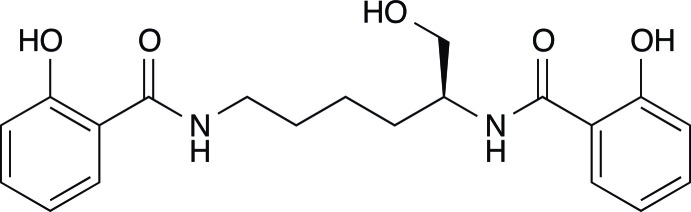


## Experimental

### 

#### Crystal data

C_20_H_24_N_2_O_5_
*M* *_r_* = 372.41
Monoclinic, 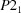

*a* = 9.5934 (7) Å
*b* = 9.2266 (7) Å
*c* = 10.3565 (7) Å
β = 96.172 (4)°
*V* = 911.39 (11) Å^3^
*Z* = 2
Mo *K*α radiation
μ = 0.10 mm^−1^
*T* = 123 K
0.26 × 0.21 × 0.04 mm

#### Data collection

Bruker Nonius X8 APEX diffractometer
Absorption correction: multi-scan (*SADABS*; Bruker, 2010 ▶) *T*_min_ = 0.975, *T*_max_ = 0.996
10366 measured reflections
2111 independent reflections
1943 reflections with *I* > 2σ(*I*)
*R*_int_ = 0.028

#### Refinement

*R*[*F* ^2^ > 2σ(*F* ^2^)] = 0.031
*wR*(*F* ^2^) = 0.076
*S* = 1.05
2111 reflections
259 parameters
6 restraints
H atoms treated by a mixture of independent and constrained refinement
Δρ_max_ = 0.21 e Å^−3^
Δρ_min_ = −0.22 e Å^−3^

### 

Data collection: *APEX2* (Bruker, 2010 ▶); cell refinement: *SAINT* (Bruker, 2010 ▶); data reduction: *SAINT*; program(s) used to solve structure: *SHELXS97* (Sheldrick, 2008 ▶); program(s) used to refine structure: *SHELXL97* (Sheldrick, 2008 ▶); molecular graphics: *DIAMOND* (Brandenburg, 2012 ▶); software used to prepare material for publication: *SHELXL97*.

## Supplementary Material

Crystal structure: contains datablock(s) global, I. DOI: 10.1107/S1600536813000354/yk2084sup1.cif

Structure factors: contains datablock(s) I. DOI: 10.1107/S1600536813000354/yk2084Isup2.hkl

Supplementary material file. DOI: 10.1107/S1600536813000354/yk2084Isup3.cml

Additional supplementary materials:  crystallographic information; 3D view; checkCIF report

## Figures and Tables

**Table 1:** Hydrogen-bond geometry (Å, °)

*D*—H⋯*A*	*D*—H	H⋯*A*	*D*⋯*A*	*D*—H⋯*A*
O6—H6O⋯O7	0.88 (2)	1.70 (2)	2.530 (2)	155 (3)
N8—H8N⋯O14^i^	0.87 (2)	2.21 (2)	3.051 (2)	163 (2)
O14—H14O⋯O16^ii^	0.85 (2)	2.03 (2)	2.858 (2)	165 (3)
N15—H15N⋯O7^iii^	0.86 (2)	2.25 (2)	3.046 (2)	154 (2)
O22—H22O⋯O16	0.84 (2)	1.95 (2)	2.648 (2)	140 (3)
O22—H22O⋯O6^iv^	0.84 (2)	2.19 (2)	2.776 (2)	127 (2)

Symmetry codes: (i) 
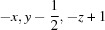
; (ii) 
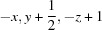
; (iii) 
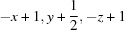
; (iv) 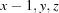.

**Fig. 1.**
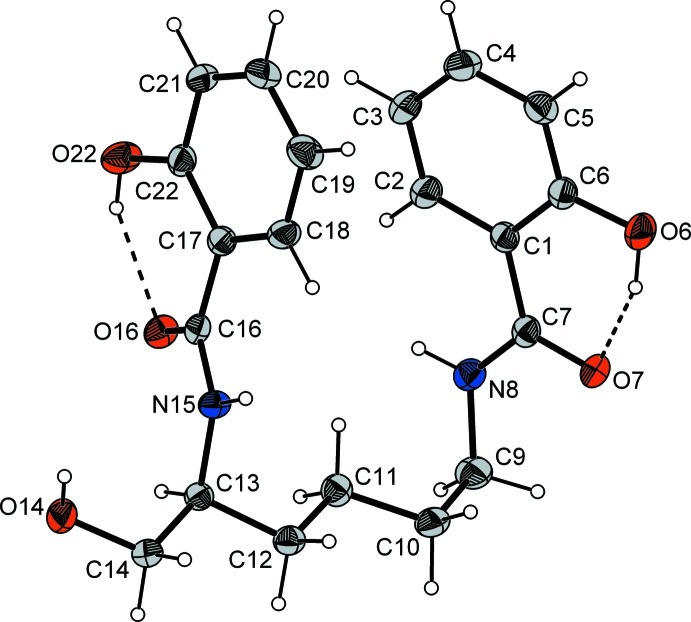
Ellipsoid plot (50% probability level) and numbering scheme of the title compound.

**Fig. 2.**
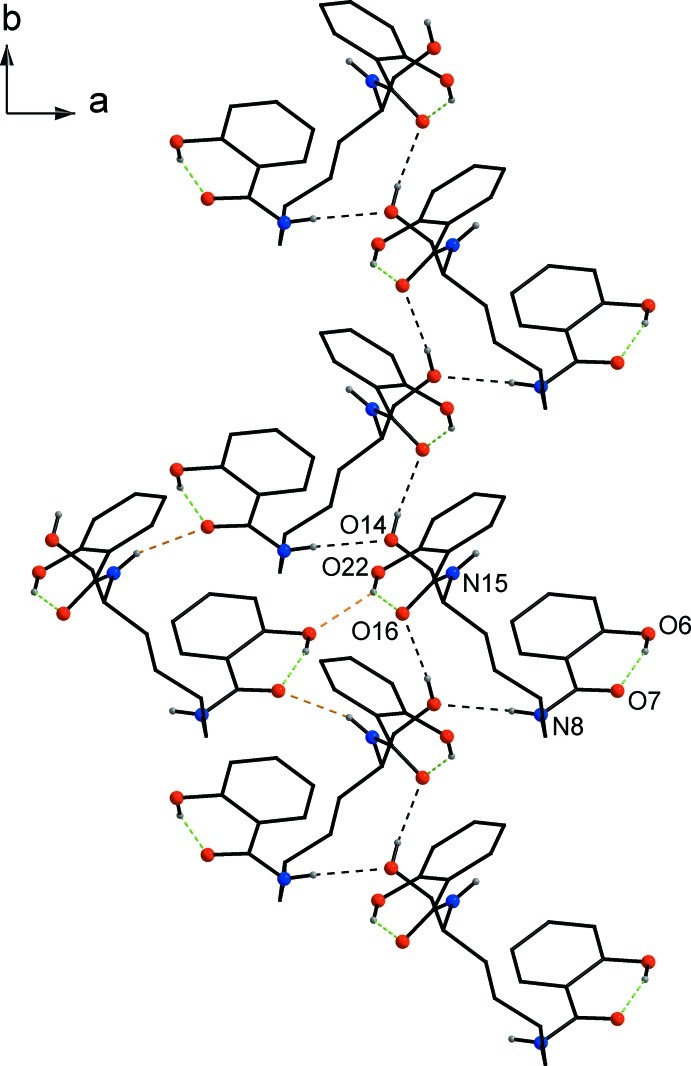
Section of a strand generated by a 21 screw (with intra- and intermolecular hydrogen bonds shown as green and black dashed lines, respectively); interlinking of such strands by additional N—H···O(amide) and O(phenolic)—H···O(phenolic) hydrogen bonding is indicated by yellow dashed lines. C atoms (black) are shown as a stick model; O (red), N (blue), H(—O) and H(—N) atoms are shown as spheres of arbitrary size. H(—C) atoms are omitted for clarity.

## supplementary crystallographic information

### Comment

Myxochelin A ((*S*)-*N*,*N*'-(6-hydroxyhexane-1,5-diyl)bis(2,3-dihydroxybenzamide)) belongs to the family of siderophores (Kunze *et al.*, 1989) and is well known for its selective complex formation with iron(III). In a neutral medium, metal binding probably occurs *via* the two catecholate groups (Huang *et al.*, 1995; Duhme *et al.*, 1996). However, myxochelin A also forms stable ferric complexes in an acidic medium around pH 2–3. Under such conditions, a complete deprotonation of all four phenolic hydroxy groups is unfavourable and, alternatively, metal binding may rather occur in a bis-bidentate fashion *via* the two *ortho*-hydroxy-benzamide moieties (Cappillino *et al.*, 2009; Stoicescu *et al.*, 2009). For a direct investigation of such a coordination mode, we prepared dideoxy-myxochelin A as a model ligand and report here its crystal structure. The structure elucidation of a related achiral derivative, which is devoid of the hydroxymethyl group, has been reported by Huang *et al.* (1995).

The crystal structure of the title compound exhibits an all-staggered zigzag arrangement of the O14—C14—C13—C12—C11—C10—C9 chain with corresponding torsional angles of 172–179°. With regard to this chain, the two *ortho*-hydroxybenzamide moieties adopt a *gauche* conformation with C—C—C—N torsional angles of 54.2 (2) and -63.9 (2)°. The two phenyl rings are aligned roughly parallel (the angle between the two mean planes is 23°). Inspection of interatomic distances revealed, however, that the interaction between the two aromatic moieties should be interpreted in terms of simple van der Waals contacts rather than π–π stacking. The amide groups and the corresponding phenyl rings are almost, but not fully, coplanar. The angle between the mean planes defined by C1–C6 and N8, C7, O7, C1 or C17–C22 and N15, C16, O16, C17 is 5° or 19°, respectively. Both aromatic hydroxy groups are involved in intramolecular O—H···O(carbonyl) hydrogen bonding, forming a six-membered ring structure which is quite often observed for salicylamides. Additionally, the aliphatic hydroxy group (O14) donates its proton to the carbonyl O atom O16 of a neighbour, and the amide moiety N8—H8N donates its proton to a further aliphatic hydroxy group (O14). The two types of interactions occur along 2_1_ screws and result in the formation of infinite chains, aligned parallel to the crystallographic *b* axis. Interlinking of these chains occurs *via* N15—H15N···O7 bonding. In addition, H22O is bifurcated; beside the above-mentioned intramolecular O22—H22O···O16 bond, an intermolecular O22—H22O···O6 bond generates further interlinking along the crystallographic *a* axis. Altogether, the various types of intermolecular hydrogen bonding interactions resulted in the generation of layers, oriented parallel to the *ab* plane. Between these layers, only weak van der Waals contacts can be observed.

### Experimental

2-Hydroxybenzoic acid was allowed to react with benzyl bromide in acetone yielding 2-benzyloxy-benzoic acid, which was further transformed into (*S*)-2,6-bis(2-benzoxybenzamido)-hexan-1-ol in a two-step procedure, using thionyl chloride and subsequently (*S*)-2,6-diaminohexan-1-ol. The protecting benzoxy groups were then removed with ammonium formate and Pd/C. Off-white single crystals were grown from MeOH. Elemental analysis calculated for C_20_H_24_N_2_O_5_ (%): C 64.50, H 6.50, N 7.52; found (%): C 64.19, H 6.35, N 7.50. ^1^H NMR (DMSO-d_6_): δ (p.p.m.) = 1.38 (m, 2H), 1.62 (m, 4H), 3.30 (dt, 2H), 3.48 (m, 2H), 4.04 (m, 1H), 6.89 (m, 4H), 7.40 (m, 2H), 7.84 (dd, 1H), 7.94 (dd, 1H), 8.47 (d, NH), 8.85 (t, NH). ^13^C NMR (DMSO-d_6_): δ (p.p.m.) = 23.1, 28.7, 30.1, 38.8, 51.0, 62.9, 115.0, 115.4, 117.2, 117.3, 118.3, 127.4, 127.9, 133.4, 133.5, 160.0, 160.2.

### Refinement

In accordance with the use of the chiral and enantiomerically pure (*S*)-2,6-diaminohexan-1-ol as one of the starting materials, the title compound crystallized in a Sohncke space group. The structure contains, however, only H, C, N and O atoms and an assignment of an absolute structure was thus not possible. Therefore, a total of 1572 Friedel pairs were merged prior to the refinement and the *S*-configuration of the diaminohexanol was adopted to the title compound. All H atoms could be located. They were treated as recommended by Müller *et al.* (2006); a riding model was used for H(—C) atoms. The positional parameters of the O- and N-bonded H atoms were refined using isotropic displacement parameters, which were set to 1.5*U*_eq_ or 1.2*U*_eq_ of the pivotal O or N atom, respectively. In addition, restraints of 0.84 and 0.88 Å were used for the O—H and N—H distances.

### Crystal data

**Table:**

C_20_H_24_N_2_O_5_	*F*(000) = 396
*M**_r_* = 372.41	*D*_x_ = 1.357 Mg m^−^^3^
Monoclinic, *P*2_1_	Mo *K*α radiation, λ = 0.71073 Å
Hall symbol: P 2yb	Cell parameters from 4681 reflections
*a* = 9.5934 (7) Å	θ = 2.8–29.3°
*b* = 9.2266 (7) Å	µ = 0.10 mm^−^^1^
*c* = 10.3565 (7) Å	*T* = 123 K
β = 96.172 (4)°	Prism, light brown
*V* = 911.39 (11) Å^3^	0.26 × 0.21 × 0.04 mm
*Z* = 2	

### Data collection

**Table:**

Bruker Nonius X8 APEX diffractometer	2111 independent reflections
Radiation source: fine-focus sealed tube	1943 reflections with *I* > 2σ(*I*)
Graphite monochromator	*R*_int_ = 0.028
Detector resolution: 8.4 pixels mm^-1^	θ_max_ = 27.0°, θ_min_ = 2.0°
φ and ω scans	*h* = −12→11
Absorption correction: multi-scan (*SADABS*; Bruker, 2010)	*k* = −11→9
*T*_min_ = 0.975, *T*_max_ = 0.996	*l* = −13→13
10366 measured reflections	

### Refinement

**Table:**

Refinement on *F*^2^	Primary atom site location: structure-invariant direct methods
Least-squares matrix: full	Secondary atom site location: difference Fourier map
*R*[*F*^2^ > 2σ(*F*^2^)] = 0.031	Hydrogen site location: inferred from neighbouring sites
*wR*(*F*^2^) = 0.076	H atoms treated by a mixture of independent and constrained refinement
*S* = 1.05	*w* = 1/[σ^2^(*F*_o_^2^) + (0.0425*P*)^2^ + 0.1259*P*] where *P* = (*F*_o_^2^ + 2*F*_c_^2^)/3
2111 reflections	(Δ/σ)_max_ < 0.001
259 parameters	Δρ_max_ = 0.21 e Å^−^^3^
6 restraints	Δρ_min_ = −0.22 e Å^−^^3^

### Special details

**Table:**

Geometry. All e.s.d.'s (except the e.s.d. in the dihedral angle between two l.s. planes) are estimated using the full covariance matrix. The cell e.s.d.'s are taken into account individually in the estimation of e.s.d.'s in distances, angles and torsion angles; correlations between e.s.d.'s in cell parameters are only used when they are defined by crystal symmetry. An approximate (isotropic) treatment of cell e.s.d.'s is used for estimating e.s.d.'s involving l.s. planes.
Refinement. Refinement of *F*^2^ against ALL reflections. The weighted *R*-factor *wR* and goodness of fit *S* are based on *F*^2^, conventional *R*-factors *R* are based on *F*, with *F* set to zero for negative *F*^2^. The threshold expression of *F*^2^ > σ(*F*^2^) is used only for calculating *R*-factors(gt) *etc*. and is not relevant to the choice of reflections for refinement. *R*-factors based on *F*^2^ are statistically about twice as large as those based on *F*, and *R*-factors based on ALL data will be even larger.

### Fractional atomic coordinates and isotropic or equivalent isotropic displacement parameters (Å^2^)

**Table:**

	*x*	*y*	*z*	*U*_iso_*/*U*_eq_	
C1	0.4529 (2)	0.4184 (2)	0.20414 (18)	0.0199 (4)	
C2	0.3185 (2)	0.4340 (2)	0.13843 (19)	0.0258 (5)	
H2	0.2437	0.3768	0.1637	0.031*	
C3	0.2930 (2)	0.5314 (3)	0.0374 (2)	0.0313 (5)	
H3	0.2012	0.5410	−0.0062	0.038*	
C4	0.4021 (2)	0.6157 (3)	−0.0004 (2)	0.0288 (5)	
H4	0.3841	0.6832	−0.0694	0.035*	
C5	0.5358 (2)	0.6018 (3)	0.06145 (19)	0.0254 (5)	
H5	0.6098	0.6594	0.0352	0.030*	
C6	0.5621 (2)	0.5026 (2)	0.16297 (19)	0.0210 (4)	
O6	0.69536 (15)	0.49364 (19)	0.22033 (15)	0.0296 (4)	
H6O	0.685 (3)	0.441 (3)	0.290 (2)	0.044*	
C7	0.4830 (2)	0.3244 (2)	0.32048 (18)	0.0203 (4)	
O7	0.60410 (15)	0.32025 (17)	0.38085 (14)	0.0273 (4)	
N8	0.37834 (19)	0.2474 (2)	0.36263 (16)	0.0230 (4)	
H8N	0.2929 (18)	0.259 (3)	0.325 (2)	0.028*	
C9	0.3912 (2)	0.1825 (3)	0.4921 (2)	0.0284 (5)	
H9A	0.4837	0.1345	0.5091	0.034*	
H9B	0.3178	0.1078	0.4960	0.034*	
C10	0.3766 (2)	0.2972 (2)	0.59675 (19)	0.0259 (5)	
H10A	0.3778	0.2490	0.6823	0.031*	
H10B	0.4577	0.3640	0.6009	0.031*	
C11	0.2419 (2)	0.3837 (2)	0.56963 (19)	0.0228 (4)	
H11A	0.1620	0.3153	0.5593	0.027*	
H11B	0.2440	0.4355	0.4861	0.027*	
C12	0.2160 (2)	0.4936 (2)	0.67388 (18)	0.0213 (4)	
H12A	0.3007	0.5545	0.6932	0.026*	
H12B	0.1994	0.4417	0.7545	0.026*	
C13	0.0901 (2)	0.5910 (2)	0.63192 (18)	0.0192 (4)	
H13	0.0072	0.5277	0.6059	0.023*	
C14	0.0553 (2)	0.6883 (2)	0.74253 (18)	0.0225 (4)	
H14A	0.0368	0.6270	0.8173	0.027*	
H14B	0.1377	0.7497	0.7703	0.027*	
O14	−0.06297 (15)	0.77953 (17)	0.70901 (14)	0.0272 (4)	
H14O	−0.044 (3)	0.857 (2)	0.672 (2)	0.041*	
N15	0.11840 (17)	0.6788 (2)	0.51866 (15)	0.0194 (4)	
H15N	0.187 (2)	0.739 (2)	0.527 (2)	0.023*	
C16	0.06071 (19)	0.6524 (2)	0.39742 (18)	0.0185 (4)	
O16	−0.02958 (14)	0.55555 (16)	0.37353 (13)	0.0234 (3)	
C17	0.1056 (2)	0.7449 (2)	0.29126 (18)	0.0191 (4)	
C18	0.2307 (2)	0.8247 (2)	0.30504 (19)	0.0245 (5)	
H18	0.2899	0.8195	0.3846	0.029*	
C19	0.2701 (2)	0.9106 (3)	0.2062 (2)	0.0290 (5)	
H19	0.3560	0.9627	0.2174	0.035*	
C20	0.1832 (2)	0.9204 (3)	0.09003 (19)	0.0288 (5)	
H20	0.2086	0.9811	0.0223	0.035*	
C21	0.0605 (2)	0.8422 (3)	0.07330 (19)	0.0259 (5)	
H21	0.0018	0.8491	−0.0064	0.031*	
C22	0.0211 (2)	0.7531 (2)	0.17155 (18)	0.0213 (4)	
O22	−0.10130 (15)	0.68092 (19)	0.14603 (14)	0.0306 (4)	
H22O	−0.115 (3)	0.621 (3)	0.204 (2)	0.046*	

### Atomic displacement parameters (Å^2^)

**Table:**

	*U*^11^	*U*^22^	*U*^33^	*U*^12^	*U*^13^	*U*^23^
C1	0.0203 (10)	0.0213 (10)	0.0184 (9)	0.0025 (8)	0.0027 (7)	−0.0035 (8)
C2	0.0206 (10)	0.0316 (13)	0.0246 (10)	−0.0007 (9)	−0.0001 (8)	0.0035 (10)
C3	0.0202 (10)	0.0467 (15)	0.0256 (11)	0.0020 (10)	−0.0033 (8)	0.0057 (10)
C4	0.0328 (12)	0.0306 (12)	0.0227 (10)	0.0027 (10)	0.0012 (9)	0.0049 (9)
C5	0.0249 (11)	0.0276 (12)	0.0241 (10)	−0.0028 (9)	0.0053 (8)	0.0003 (9)
C6	0.0199 (9)	0.0229 (10)	0.0199 (9)	0.0021 (8)	0.0006 (7)	−0.0051 (9)
O6	0.0168 (7)	0.0382 (9)	0.0329 (8)	−0.0023 (7)	−0.0016 (6)	0.0053 (7)
C7	0.0210 (10)	0.0190 (10)	0.0207 (9)	0.0047 (8)	0.0017 (8)	−0.0044 (8)
O7	0.0210 (8)	0.0343 (9)	0.0260 (7)	0.0070 (7)	−0.0007 (6)	0.0038 (7)
N8	0.0239 (9)	0.0221 (9)	0.0228 (8)	0.0022 (8)	0.0006 (7)	0.0003 (7)
C9	0.0353 (12)	0.0225 (11)	0.0278 (10)	0.0058 (10)	0.0053 (9)	0.0068 (9)
C10	0.0295 (11)	0.0254 (12)	0.0224 (10)	0.0040 (9)	0.0017 (8)	0.0058 (9)
C11	0.0214 (10)	0.0231 (11)	0.0238 (10)	−0.0014 (9)	0.0015 (8)	−0.0003 (9)
C12	0.0213 (10)	0.0224 (10)	0.0200 (10)	−0.0026 (9)	0.0008 (8)	0.0019 (8)
C13	0.0171 (9)	0.0217 (11)	0.0187 (9)	−0.0023 (8)	0.0016 (7)	0.0033 (8)
C14	0.0220 (10)	0.0261 (11)	0.0194 (9)	0.0018 (9)	0.0020 (8)	0.0047 (9)
O14	0.0233 (8)	0.0274 (9)	0.0313 (8)	0.0060 (7)	0.0049 (6)	0.0075 (7)
N15	0.0190 (8)	0.0212 (9)	0.0173 (8)	−0.0061 (7)	−0.0009 (6)	0.0013 (7)
C16	0.0161 (9)	0.0184 (10)	0.0206 (9)	0.0031 (8)	0.0005 (7)	0.0001 (8)
O16	0.0216 (7)	0.0227 (7)	0.0247 (7)	−0.0053 (6)	−0.0028 (6)	−0.0004 (6)
C17	0.0200 (10)	0.0194 (10)	0.0178 (9)	0.0023 (8)	0.0020 (7)	−0.0025 (8)
C18	0.0213 (10)	0.0324 (12)	0.0191 (9)	−0.0033 (9)	−0.0017 (8)	0.0026 (9)
C19	0.0263 (11)	0.0345 (13)	0.0268 (10)	−0.0073 (10)	0.0058 (9)	0.0010 (10)
C20	0.0314 (12)	0.0329 (12)	0.0232 (10)	0.0024 (10)	0.0083 (9)	0.0074 (10)
C21	0.0263 (11)	0.0338 (12)	0.0171 (9)	0.0079 (10)	0.0003 (8)	0.0018 (9)
C22	0.0178 (9)	0.0251 (11)	0.0204 (9)	0.0018 (8)	0.0003 (7)	−0.0032 (9)
O22	0.0285 (8)	0.0389 (9)	0.0223 (7)	−0.0099 (7)	−0.0060 (6)	0.0021 (7)

### Geometric parameters (Å, º)

**Table:**

C1—C2	1.399 (3)	C12—C13	1.531 (3)
C1—C6	1.406 (3)	C12—H12A	0.9900
C1—C7	1.488 (3)	C12—H12B	0.9900
C2—C3	1.381 (3)	C13—N15	1.475 (2)
C2—H2	0.9500	C13—C14	1.521 (3)
C3—C4	1.394 (3)	C13—H13	1.0000
C3—H3	0.9500	C14—O14	1.425 (2)
C4—C5	1.377 (3)	C14—H14A	0.9900
C4—H4	0.9500	C14—H14B	0.9900
C5—C6	1.396 (3)	O14—H14O	0.846 (18)
C5—H5	0.9500	N15—C16	1.339 (2)
C6—O6	1.353 (2)	N15—H15N	0.858 (16)
O6—H6O	0.882 (17)	C16—O16	1.251 (2)
C7—O7	1.259 (2)	C16—C17	1.492 (3)
C7—N8	1.341 (3)	C17—C18	1.402 (3)
N8—C9	1.462 (3)	C17—C22	1.408 (3)
N8—H8N	0.874 (16)	C18—C19	1.379 (3)
C9—C10	1.532 (3)	C18—H18	0.9500
C9—H9A	0.9900	C19—C20	1.391 (3)
C9—H9B	0.9900	C19—H19	0.9500
C10—C11	1.519 (3)	C20—C21	1.376 (3)
C10—H10A	0.9900	C20—H20	0.9500
C10—H10B	0.9900	C21—C22	1.392 (3)
C11—C12	1.521 (3)	C21—H21	0.9500
C11—H11A	0.9900	C22—O22	1.351 (2)
C11—H11B	0.9900	O22—H22O	0.838 (18)
			
C2—C1—C6	118.24 (18)	C11—C12—H12A	109.2
C2—C1—C7	122.86 (18)	C13—C12—H12A	109.2
C6—C1—C7	118.77 (17)	C11—C12—H12B	109.2
C3—C2—C1	120.9 (2)	C13—C12—H12B	109.2
C3—C2—H2	119.5	H12A—C12—H12B	107.9
C1—C2—H2	119.5	N15—C13—C14	110.40 (17)
C2—C3—C4	119.94 (19)	N15—C13—C12	109.94 (15)
C2—C3—H3	120.0	C14—C13—C12	111.31 (15)
C4—C3—H3	120.0	N15—C13—H13	108.4
C5—C4—C3	120.5 (2)	C14—C13—H13	108.4
C5—C4—H4	119.8	C12—C13—H13	108.4
C3—C4—H4	119.8	O14—C14—C13	113.51 (15)
C4—C5—C6	119.7 (2)	O14—C14—H14A	108.9
C4—C5—H5	120.2	C13—C14—H14A	108.9
C6—C5—H5	120.2	O14—C14—H14B	108.9
O6—C6—C5	117.14 (18)	C13—C14—H14B	108.9
O6—C6—C1	122.18 (18)	H14A—C14—H14B	107.7
C5—C6—C1	120.67 (18)	C14—O14—H14O	114.1 (18)
C6—O6—H6O	102.2 (17)	C16—N15—C13	123.58 (17)
O7—C7—N8	120.36 (18)	C16—N15—H15N	116.8 (15)
O7—C7—C1	120.57 (18)	C13—N15—H15N	118.5 (15)
N8—C7—C1	119.04 (17)	O16—C16—N15	121.54 (18)
C7—N8—C9	121.54 (18)	O16—C16—C17	120.79 (17)
C7—N8—H8N	119.3 (16)	N15—C16—C17	117.65 (17)
C9—N8—H8N	116.1 (16)	C18—C17—C22	117.82 (18)
N8—C9—C10	111.17 (18)	C18—C17—C16	122.51 (16)
N8—C9—H9A	109.4	C22—C17—C16	119.67 (17)
C10—C9—H9A	109.4	C19—C18—C17	121.74 (18)
N8—C9—H9B	109.4	C19—C18—H18	119.1
C10—C9—H9B	109.4	C17—C18—H18	119.1
H9A—C9—H9B	108.0	C18—C19—C20	119.5 (2)
C11—C10—C9	111.91 (17)	C18—C19—H19	120.2
C11—C10—H10A	109.2	C20—C19—H19	120.2
C9—C10—H10A	109.2	C21—C20—C19	120.0 (2)
C11—C10—H10B	109.2	C21—C20—H20	120.0
C9—C10—H10B	109.2	C19—C20—H20	120.0
H10A—C10—H10B	107.9	C20—C21—C22	120.90 (18)
C10—C11—C12	114.81 (16)	C20—C21—H21	119.5
C10—C11—H11A	108.6	C22—C21—H21	119.5
C12—C11—H11A	108.6	O22—C22—C21	116.52 (17)
C10—C11—H11B	108.6	O22—C22—C17	123.48 (18)
C12—C11—H11B	108.6	C21—C22—C17	119.97 (19)
H11A—C11—H11B	107.5	C22—O22—H22O	112.6 (19)
C11—C12—C13	111.96 (16)		
			
C6—C1—C2—C3	−1.2 (3)	C11—C12—C13—C14	173.46 (16)
C7—C1—C2—C3	174.6 (2)	N15—C13—C14—O14	58.6 (2)
C1—C2—C3—C4	0.1 (3)	C12—C13—C14—O14	−178.98 (16)
C2—C3—C4—C5	0.6 (4)	C14—C13—N15—C16	−131.63 (19)
C3—C4—C5—C6	−0.1 (3)	C12—C13—N15—C16	105.2 (2)
C4—C5—C6—O6	−179.9 (2)	C13—N15—C16—O16	4.9 (3)
C4—C5—C6—C1	−1.0 (3)	C13—N15—C16—C17	−176.42 (17)
C2—C1—C6—O6	−179.51 (19)	O16—C16—C17—C18	−161.58 (19)
C7—C1—C6—O6	4.5 (3)	N15—C16—C17—C18	19.7 (3)
C2—C1—C6—C5	1.7 (3)	O16—C16—C17—C22	17.9 (3)
C7—C1—C6—C5	−174.34 (18)	N15—C16—C17—C22	−160.80 (19)
C2—C1—C7—O7	−175.9 (2)	C22—C17—C18—C19	1.0 (3)
C6—C1—C7—O7	0.0 (3)	C16—C17—C18—C19	−179.5 (2)
C2—C1—C7—N8	2.1 (3)	C17—C18—C19—C20	0.8 (3)
C6—C1—C7—N8	177.93 (19)	C18—C19—C20—C21	−1.5 (3)
O7—C7—N8—C9	13.5 (3)	C19—C20—C21—C22	0.2 (3)
C1—C7—N8—C9	−164.46 (18)	C20—C21—C22—O22	179.86 (19)
C7—N8—C9—C10	75.7 (2)	C20—C21—C22—C17	1.6 (3)
N8—C9—C10—C11	54.2 (2)	C18—C17—C22—O22	179.7 (2)
C9—C10—C11—C12	176.49 (17)	C16—C17—C22—O22	0.2 (3)
C10—C11—C12—C13	172.07 (17)	C18—C17—C22—C21	−2.2 (3)
C11—C12—C13—N15	−63.9 (2)	C16—C17—C22—C21	178.28 (18)

### Hydrogen-bond geometry (Å, º)

**Table:**

*D*—H···*A*	*D*—H	H···*A*	*D*···*A*	*D*—H···*A*
O6—H6*O*···O7	0.88 (2)	1.70 (2)	2.530 (2)	155 (3)
N8—H8*N*···O14^i^	0.87 (2)	2.21 (2)	3.051 (2)	163 (2)
O14—H14*O*···O16^ii^	0.85 (2)	2.03 (2)	2.858 (2)	165 (3)
N15—H15*N*···O7^iii^	0.86 (2)	2.25 (2)	3.046 (2)	154 (2)
O22—H22*O*···O16	0.84 (2)	1.95 (2)	2.648 (2)	140 (3)
O22—H22*O*···O6^iv^	0.84 (2)	2.19 (2)	2.776 (2)	127 (2)

Symmetry codes: (i) −*x*, *y*−1/2, −*z*+1; (ii) −*x*, *y*+1/2, −*z*+1; (iii) −*x*+1, *y*+1/2, −*z*+1; (iv) *x*−1, *y*, *z*.
